# MicroRNAs in metamorphic and non-metamorphic transitions in hemimetabolan insect metamorphosis

**DOI:** 10.1186/1471-2164-13-386

**Published:** 2012-08-10

**Authors:** Mercedes Rubio, Anibal de Horna, Xavier Belles

**Affiliations:** 1 Institute of Evolutionary Biology (CSIC-UPF), Passeig Marítim 39, 08003 Barcelona, Spain

**Keywords:** MicroRNAs, Metamorphosis, Ecdysone, Juvenile hormone, *Blattella*, Cockroach, *Drosophila*

## Abstract

**Background:**

Previous work showed that miRNAs play key roles in the regulation of metamorphosis in the hemimetabolan species *Blattella germanica*. To gain insight about which miRNAs might be important, we have constructed two miRNA libraries, one of the penultimate, pre-metamorphic nymphal instar (N5) and the other of the last, metamorphic nymphal instar (N6).

**Results:**

High throughput sequencing gave 61 canonical miRNAs present in the N5 and N6 libraries, although at different proportions in each. Comparison of both libraries led to the identification of three and 37 miRNAs significantly more expressed in N5 and N6 respectively. Twelve of these 40 miRNAs were then investigated further by qRT-PCR and results indicated that miR-252-3p was well expressed in N5 but not in N6, whereas let-7-5p, miR-100-5p and miR-125-5p showed the reverse pattern. 20-Hydroxyecdysone (20E) tended to stimulate miRNA expression, whereas juvenile hormone (JH) inhibited the 20E stimulatory effect. Expression of let-7, miR-100 and miR-125 was increased by 20E, which has also been observed in *D. melanogaster*. The only miRNA that was inhibited by 20E was miR-252-3p. The involvement of let-7, miR-100 and miR-125 in metamorphosis has been demonstrated in other insects. Depletion of miR-252-3p caused growth and developmental delays, which suggests that this miRNA is involved in regulating these processes prior to metamorphosis.

**Conclusions:**

The comparative analysis of miRNA libraries from pre-metamorphic (N5) and metamorphic stages (N6) of *B. germanica* proved to be a useful tool to identify miRNAs with roles in hemimetabolan metamorphosis. Three miRNAs emerged as important factors in the metamorphic stage (N6): let-7-5p, miR-100-5p and miR-125-5p, whereas miR-252-3p appears to be important in the pre-metamorphic stage (N5).

## Background

Metamorphosis has been a key innovation in insect evolution, which had a decisive influence on the dramatic diversification of this animal class, with around one million presently described species [1]. There are two types of metamorphosis: hemimetaboly, or gradual metamorphosis, and holometaboly or abrupt metamorphosis [2], the latter being evolutionarily the most successful [1]. In all cases, insect metamorphosis is hormonally regulated, and the most important hormones are the ecdysteroid 20-hydroxyecdysone (20E), which determines the successive moults, and the terpenoid juvenile hormone (JH), which represses the expression of adult features. Each hormone exerts its respective action through a cascade of transcription factors that transduce the hormonal signal to the effector genes [3,4].

Recently, a number of reports have pointed to microRNAs (miRNAs) as important players in the metamorphic transition, in hemimetabolan as well as in holometabolan species [5]. miRNAs are small non-coding RNAs of about 21–22 nucleotides that modulate gene expression at a post-transcriptional level, often in the context of developmental and morphogenetic processes [6-8]. They undergo molecular processing before becoming mature miRNAs [9,10]. First, miRNAs are transcribed as part of a primary transcript (pri-miRNA), which contains one or more miRNA precursors (pre-miRNAs). In the nucleus, the pri-miRNAs are processed into hairpin-structured pre-miRNAs by the ribonuclease Drosha, and exported to the cytoplasm. Once in the cytoplasm, the pre-miRNAs are cleaved by the ribonuclease Dicer-1 into an imperfectly-paired duplex, whose 5’- and 3’-strands can either give two respective miRNAs or only one. Generally, only one of the strands give rise to a mature miRNA, whereas the other, called miRNA* or the star strand, is degraded [9]. However, given that both strands can give mature miRNAs in some cases [11], the miRNAs are presently labelled as miRNA-5p or miRNA-3p according to the position of the original strand in the pre-miRNA. This is the convention used in the present paper.

One of the more dramatic demonstrations that miRNAs are involved in insect metamorphosis was reported by Gomez-Orte & Belles [12], who silenced *dicer-1* expression by RNAi in the last nymphal instar of the cockroach *Blattella germanica*, and obtained supernumerary nymphs after the following moult, instead of adults [12]. These results showed that Dicer-1 and miRNAs are important factors in the mechanisms regulating metamorphosis. However, which particular miRNAs were involved remained unclear.

*B. germanica* is a polyneopteran exopterigote insect that shows a gradual morphological transformation during the life cycle, and is a good representative of the less modified hemimetaboly. This, and the fact that it is especially sensitive to gene silencing by RNAi [13], have made *B. germanica* a favourite hemimetabolan model to study metamorphosis. However, the genome of *B. germanica* is yet to be sequenced. The first step to approach the study of miRNAs involved in metamorphosis was therefore to establish a baseline miRNA catalogue using high-throughput sequencing [14]. To gain more information about which miRNAs may be important for *B. germanica* metamorphosis, we constructed two miRNA libraries, one with RNA extracted around the peak of 20E of the penultimate nymphal instar (N5), and the other constructed equivalently but during the last instar (N6). The endocrine context differs between these two libraries with the presence of JH in N5 and its absence in N6. We therefore aimed to identify miRNAs differentially expressed in N5 and N6. Those that were identified with this approach were further studied by establishing the expression pattern in N5 and N6, and by investigating the effect of 20E alone and that of 20E plus JH in their expression. Finally, we selected miR-252-3p, the most interesting miRNA emerging from these studies, to carry out a functional analysis using a specific anti-miRNA.

## Methods

### Insects

Specimens of *B. germanica* were obtained from a colony reared in the dark at 30 ± 1°C and 60–70% relative humidity. Freshly ecdysed nymphs or adult females were selected and used at the appropriate ages. All dissections, treatments and tissue sampling were carried out on carbon dioxide-anaesthetized specimens. Samples for RNA extraction and quantification were frozen immediately after dissection, and stored at −80°C until use.

### Samples to construct the miRNA libraries

To construct the two miRNA libraries, we carried out total RNA extraction from the whole body (excluding the head and the digestive tube, to avoid ocular pigments and intestine parasites) of *B. germanica* specimens, using the miRNeasy extraction kit (QIAGEN). Nine individuals taken in days 3, 4 and 5 (three each day) of fifth nymphal instar were pooled for the N5 library. The equivalent sampling was carried out on days 5, 6 and 7 of sixth (last) nymphal instar for the N6 library. The days chosen correspond to the 20E respective moulting peaks in N5 and N6 of *B. germanica*[15].

### Analysis of miRNA libraries

Solexa technology (using an Illumina HiSeq 2000 sequencer) was used for small RNA sequencing in *B. germanica*. Sequences were received in FASTQ format, with the adaptor sequences trimmed. The first filter applied was based on quality values; considering that Illumina quality values range from 0 to 40 (using ASCII 64–104 in fastq) [16], we eliminated those reads having 80% of the sequence with values lower than 20. Then we applied a trimming process to delete regions with repetitive nucleotides or short motifs, and low quality regions, either at the beginning or at the end of the sequence. Sequences with the same length were grouped, then we obtained the counts collapsing the sequences using the suite of programs Fastx-toolkit (http://hannonlab.cshl.edu/fastx_toolkit), and those sequences with 10–28 nucleotides were kept. To eliminate artefacts, we mapped the sequences against Pfam database (excluding miRNAs), and against the genome sequences of *Blattabacterium cuenoti*, a bacteroid endosimbiont of *B. germanica*[17], *B. germanica* densovirus [18] and *Escherichia coli*. The remaining sequences were considered putative miRNAs. We identified the canonical miRNAs by Bowtie alignment [19] against mature miRNAs of the mirBASE database (November 2011 version) [20]. We followed the -3p or -5p nomenclature, instead of mature and star strand, assuming that the miRNA position in the precursor of *B. germanica* was conserved with respect to *D. melanogaster*. The seed was defined as the first 17 nucleotides and not mismatches were allowed [21]. Sequences shorter than 17 nucleotides were labelled as a given miRNA when they had 100% matching with the corresponding region of the miRNA seed. For expression analysis, different miRNAs length variants, or isoMIRs [22] of the same miRNA were consider together. Finally, the poorest represented miRNAs, i.e., the pool of those representing only 0.01% of the total reads, were eliminated from the differential expression analysis.

### Comparison of miRNA libraries

In order to compare the N5 and N6 libraries, the counts were normalized by transforming them into RPKM (Reads Per Kilobase Mapped) [23] and were analysed by the NOISeq R package [24]. The counts of the different miRNA length variants were merged. The method of NOISeq applied was NOISeqsim, which assumes that there are not replicates for the experimental conditions and it simulates them. We used the default values to define those miRNAs differentially expressed (probability > 0.8). The evaluation of the results was based on the NOISeq scores: probability of being differentially expressed, M-value and D-value. The results were represented by a Volcano plot, as described by Cui and Churchill [25].

### Expression patterns

miRNA expression patterns were established at 48 h intervals in female specimens of N5 and N6 and in freshly emerged adults. Three biological replicates of each age were studied. Whole body sampling and total RNA extraction was carried out as in the library construction. Quantification of miRNA levels was carried out by quantitative real-time PCR (qRT-PCR). An amount of 400 ng of total RNA was reverse transcribed with the NCode miRNA first-strand synthesis and qRT-PCR kit (Invitrogen), following the manufacturer protocol. Amplification reactions were carried out using IQTM SYBR Green Supermix (BioRad) and the following protocol: 95°C for 2 min, and 40 cycles of 95°C for 15 s and 60°C for 30 s, in a MyIQ Real-Time PCR Detection System (BioRad). A dissociation curve was obtained to ensure that there was only one product amplified after the amplification phase. All reactions were run in triplicate. The endogenous reference gene used was U6, as in [26]; therefore, results are given as copies of RNA per 1000 copies of U6. Primer sequences for each miRNA are available on request.

### Hormone treatments

In a first set of experiments, specimens were treated as freshly emerged sixth instar nymph (N6), by injecting 1 μL of 20E (Sigma) at a concentration of 1 μg/μL dissolved in water with 10% ethanol; controls where treated equivalently with 1 μL of water with 10% ethanol. Injection was performed through the membrane between fourth and fifth ventrites. In another experimental set, specimens were treated at the same stage, by applying topically 1 μL of JH III (Sigma) dissolved in acetone at a concentration of 2 μg/μL, and with 20E in the same conditions as in the previous set of experiments; controls were treated equivalently with 1 μL of acetone and 1 μL of water with 10% ethanol. JH III is the native JH of *B. germanica*[27], and given that the commercial JH III is a mixture of isomers containing ca. 50% of the biologically active (10*R*)-JH III, thus the active dose applied was around 1 μg per specimen. Total RNA extraction and quantification of *dicer-1* expression and miRNAs were performed 24 h after the treatment following the procedures described above, and using three biological replicates. Statistical analysis of relative expression measurements was carried out with the REST software tool [28].

### miRNA depletion

To deplete miR-252-3p expression we used miRCURY LNA™ microRNA Power Inhibitor (Exiqon). The sequence of LNA anti-miR-252-3p (GATAAGCACTTGAGCAGCAGG) is quite similar to the sequence of miR-252-5p (CTAAGTACTAGTGCCGCAGGA), which suggests that it might act as miR-252-5p mimic. However, the seed of LNA-antimiR-252-3p (ATAAGC) is very different to that of miR-252-5p (TAAGTA), which makes very improbable this possibility. A dose of 1 μL of LNA at 50 μM was injected in the abdominal cavity 24 h after the moult to the fifth instar nymph (N5), and a second injection at the same concentration was carried out 48 h later. Preliminary experiments administering a single dose 24 h after the moult to N5 depleted miR-252-3p less efficiently. Controls were injected with miRCURY LNA™ microRNA Inhibitor Negative Control A (Exiqon) at the same stages and concentrations. Specimens for miRNA quantification were collected 24 h after the second injection. RNA extraction, quantification of miRNA levels and statistical analysis were performed as described above. Specimens to study the phenotype were left alive until the adult moult.

## Results

### Comparison of libraries and miRNA selection for further studies

**Figure 1:**
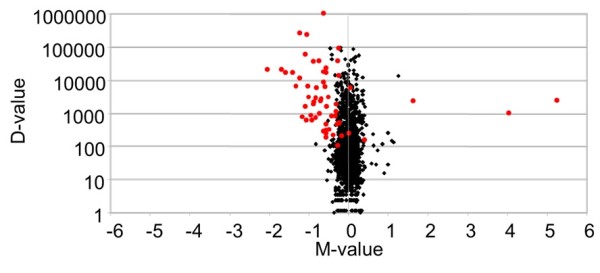
**Volcano plot showing differentially expressed miRNAs in N5 and N6 libraries of *Blattella germanica*.** M-D values in noise are represented by black dots, and differentially expressed miRNAs by red dots. M-value: log_2_(x_1_/x_2_). D-value: |x_1_-x_2_|. Where *x*_*i*_ are the expression level in RPKMs in library *i*. The more external points represent the more differentially expressed values, and correspond to the miRNA analyzed.

The sequencing of the N5 and N6 miRNA libraries of *B. germanica* resulted in 38,399,972 and 23,689,204 reads respectively, with sequence lengths between 10 and 40 nucleotides. From these, the application of the filters described in the Material and Methods section led to 2,260,210 and 2,174,640 reads in the N5 and N6 libraries respectively, corresponding to 61 canonical miRNAs. The conversion of the reads of each miRNA into RPKM in the N5 and N6 libraries gave an initial insight into which miRNAs were more and less expressed in each library (Table 1). Calculation of the probability of being differentially expressed, M-value and D-value led to the identification of those miRNAs that are differentially expressed in N5 and in N6. These calculations indicate that three miRNAs are more highly expressed in N5 (those with a positive M-value; Table 2), and 37 are more highly expressed in N6 (those with a negative M-value; Table 3). Results are summarized in a Volcano plot (Figure 1).

**Table 1:** Counts and RPKM of the canonical miRNAs found in the N5 and N6 libraries

**miRNA**	**N5**		**N6**	
	**Counts**	**RPKM**	**Counts**	**RPKM**
bantam-3p	4355	5939.042	10724	24195.476
let-7-5p	6238	8608.982	10157	22754.573
miR-iab-4-5p	618	856.282	559	1253.199
miR-1-3p	1162418	1676872.803	1101990	2579427.475
miR-2-3p	16839	30473.966	20102	44802.071
miR-7-5p	1115	1603.854	764	1782.859
miR-8-3p	129951	173738.073	185336	401628.852
miR-8-5p	36142	50136.290	37104	83764.303
miR-9a-3p	1316	1810.390	990	2209.068
miR-9a-5p	7345	10519.521	6705	15734.350
miR-9b-3p	444	610.332	504	1151.966
miR-9c-5p	2436	3731.535	3748	9306.244
miR-10-3p	2018	2857.459	1542	3543.522
miR-10-5p	10308	14188.293	9746	21761.685
miR-12-5p	607	811.513	714	1543.625
miR-13a-3p	923	1268.955	946	2090.234
miR-13b-3p	2436	3124.586	2431	5064.433
miR-14-3p	89736	124156.170	52803	118969.680
miR-31-5p	5091	7007.565	5468	12172.024
miR-34-5p	3124	4465.801	2343	5457.763
miR-71-3p	5006	6984.393	3798	8620.928
miR-71-5p	31574	43472.049	28524	63933.294
miR-79-3p	186	277.628	504	1151.966
miR-87-3p	4999	7205.065	7177	16792.743
miR-92a-3p	529	722.703	450	1001.511
miR-92b-3p	1915	2632.691	1375	3066.353
miR-100-5p	5943	8211.876	11666	26310.508
miR-124-3p	406	608.142	325	792.840
miR-125-5p	5131	7347.148	9416	21901.480
miR-133-3p	1951	2682.100	1404	3130.094
miR-184-3p	106965	167335.182	85068	200902.694
miR-190-5p	2223	2898.137	440	935.079
miR-193-3p	340	472.188	319	724.041
miR-252-3p	1587	2175.501	25	56.778
miR-252-5p	20378	29676.940	19167	45269.029
miR-263a-5p	30064	38608.305	34005	70511.600
miR-263b-5p	1470	1918.685	1669	3538.234
miR-275-3p	6538	19349.775	8708	19144.342
miR-276-3p	133114	192096.386	176820	394590.630
miR-276-5p	662	902.984	28	54.987
miR-277-3p	3881	5345.294	4880	10875.555
miR-278-3p	588	851.690	636	1492.383
miR-279-3p	33581	47196.919	43314	99043.811
miR-281-3p	346	479.946	442	1001.721
miR-281-5p	2412	3298.680	2637	5834.284
miR-283-5p	1684	2362.825	1891	4289.933
miR-305-5p	4620	6304.241	3952	8866.733
miR-306-5p	45159	61925.032	32982	73346.550
miR-307-3p	1829	2557.487	2247	5124.163
miR-315-5p	345	465.641	254	556.891
miR-316-5p	2143	2971.686	1923	4322.012
miR-317-3p	311250	429211.869	226595	509795.976
miR-375-3p	2744	3957.724	2693	6304.047
miR-927-5p	1167	1603.931	721	1608.971
miR-965-3p	420	554	381	816.976
miR-993-3p	371	531.079	170	398.526
miR-998-3p	242	328.263	221	484.999
miR-2765-5p	401	540.370	553	1210.667
miR-2788-3p	300	427.236	271	632.329
miR-2796-3p	903	1216.746	1172	2550.380
miR-3770-5p	1383	2086.531	1141	2800.141

**Table 2:** miRNAs differentially expressed in N5 sorted by the absolute M-value

**miRNA**	***P***	**M-value**	**D-value**
miR-252-3p	0.917	5.260	2118.72
miR-276-5p	0.852	4.037	847.99
miR-190-5p	0.912	1.632	1963.06

The higher the M-value the more differentially expressed is the miRNA. *P*: Probability of being differentially expressed.

**Table 3:** miRNAs differentially expressed in N6, sorted by the M-value

**miRNA**	***P***	**M-value**	**D-value**
bantam-3p	0.988	−2.026	18256.44
miR-100-5p	0.988	−1.680	18098.63
miR-125-5p	0.987	−1.576	14554.33
let-7-5p	0.986	−1.402	14145.59
miR-9c-5p	0.959	−1.318	5574.70
miR-87-3p	0.973	−1.221	9587.67
miR-8-3p	0.999	−1.209	227890.78
miR-2765-5p	0.830	−1.164	670.30
miR-279-3p	0.994	−1.069	51846.89
miR-2796-3p	0.884	−1.068	1333.63
miR-276-3p	0.998	−1.039	202494.24
miR-277-3p	0.956	−1.025	5530.26
miR-307-3p	0.923	−1.003	2566.67
miR-12-5p	0.837	−0.928	732.12
miR-263b-5p	0.895	−0.883	1619.55
miR-263a-5p	0.986	−0.869	31903.3
miR-283-5p	0.907	−0.860	1927.11
miR-281-5p	0.918	−0.823	2535.60
miR-278-3p	0.821	−0.809	640.69
miR-31-5p	0.951	−0.797	5164.45
miR-8-5p	0.984	−0.740	33628.01
miR-13a-3p	0.840	−0.720	821.27
miR-13b-3p	0.904	−0.697	1939.84
miR-375-3p	0.914	−0.672	2346.33
miR-1-3p	0.990	−0.622	902554.68
miR-10-5p	0.960	−0.617	7573.40
miR-252-5p	0.977	−0.609	15592.09
miR-9a-5p	0.947	−0.581	5214.83
miR-71-5p	0.977	−0.556	20461.24
miR-2-3p	0.974	−0.556	14328.10
miR-316-5p	0.878	−0.540	1350.32
miR-305-5p	0.910	−0.492	2562.49
miR-3770-5p	0.813	−0.424	713.61
miR-71-3p	0.8201	−0.304	1636.54
miR-184-3p	0.870	−0.264	33567.51
miR-317-3p	0.865	−0.248	80584.11
miR-306-5p	0.815	−0.244	11421.52

The lower M-value, the more differentially expressed is the miRNA. *P*: Probability of being differentially expressed.

Notably, both strands of the miRNA precursor miR-252 are well present in the two libraries, suggesting that both of them are biologically active miRNAs. The 5’ strand (miR-252-5p) is about 12-fold more abundant than the 3’ strand (miR-252-3p) in the N5 library and ca. 800-fold times more abundant in N6. Furthermore, from N5 to N6 there is an increase of miR-252-5p and a significant decrease of miR-252-3p (Table 1). miR-276 represents a similar case, with both strands represented in both libraries. In this case, however, the 3’ strand (miR-276-3p) is the predominant one in both libraries, being clearly more highly expressed in N6 with respect to N5, whereas the 5’ strand (miR-276-5p) is more highly expressed in the N5 with respect to N6.

For expression pattern studies and hormonal treatments, we selected the three miRNAs more highly expressed (M-value between 1.6 and 5.3) in the N5 library (miR-252-3p, miR-276-5p and miR-190-5p) and the four more differentially expressed (M-value between −1.4 and −2.0) in the N6 library (bantam-3p, miR-100-5p, miR-125-5p and let-7-5p). We also added three miRNAs that are similarly represented in N5 and N6: miR-252-5p and miR-276-3p, whose respective partner strands were chosen due to their higher expression in the N5 library, and miR-1-3p, which was the most abundant miRNA (accounting for more than 50% of total reads). Finally, we included two additional miRNAs (miR-14-3p and miR-34-5p) because of their potential interest in the context of moulting and metamorphosis. miR-14-3p has been described targeting the ecdysone receptor (EcR) in *D. melanogaster*[29], and miR-34-5p is inducible by the JH analogue methoprene in *Drosophila* S2 cells [30].

### miRNA expression patterns

**Figure 2:**
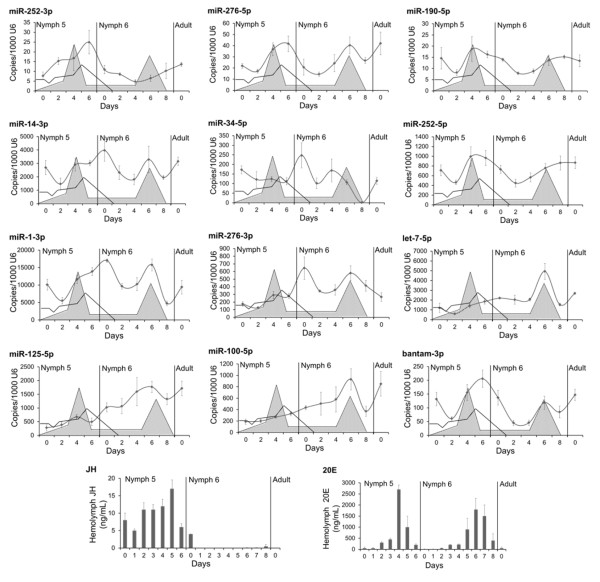
**Expression pattern of the 12 miRNA selected from the comparison of the N5 and N6 libraries in *Blattella germanica*.** miRNA levels were measured on nymph 5, nymph 6 and in freshly emerged adults with qRT-PCR; data represent the mean ± SEM, and are indicated as copies of the respective miRNA per 1000 copies of U6; each point represents 3 biological replicates. Schematic patterns of juvenile hormone III (JH) and 20-hydroxyecdysone (20E) titers in nymph 5 and nymph 6 are superimposed in every graphic as empty and grey patterns, respectively. Actual values of JH and 20E titers are represented in the two bottom panels, according to the data of Treiblmayr et al. [41] (JH) and Romaña et al. [15] (20E).

The miRNA expression patterns were determined in N5, and in N6, as well as in freshly emerged adults. One of the most peculiar patterns was that of miR-252-5p, which showed a burst of expression towards the end of N5, approximately one or two days after the peak of 20E, and then the expression steadily decreased during most of the N6, including the days of the peak of 20E of this stage (Figure 2). This pattern is consistent with counts data obtained from the bioinformatical analysis of N5 and N6. Also interesting are the patterns of let-7, miR-100 and miR-125, which show low expression in N5, which then increased in N6 reaching a burst around the days of the 20E peak (Figure 2); these patterns are also consistent with the sequencing data.

The patterns of the remaining miRNAs examined show two bursts of expression. In the cases of miR-276-5p, miR-190-5p, miR-252-5p and bantam-3p, there were expression bursts seen both in N5 and in N6, approximately corresponding to the peaks of 20E (Figure 2). These data do not correlate with quantitative sequencing data, which predicted that miR-276-5p and miR-190-5p should have shown much higher expression levels around the 20E peak of N5, whereas those of miR-252-5p and bantam-3p should have been much lower at this stage. Two expression bursts of miR-1-3p, miR-34-5p and mir-276-3p occurred in N6, the first just after emergence and the other coinciding or close to the peak of 20E of this stage (Figure 2). Comparing the levels of expression around the peaks of 20E in N5 and N6, these approximately patterns correspond to what would be expected based on the sequencing data. Minor discrepancies, like in the case of miR-276-5p, miR-276-3p, miR-190-5p, can be due to individual variability, given that sequencing data come from a pool of nine specimens, whereas data of expression patterns is based on three particular specimens.

### Effect of hormonal treatments

**Figure 3:**
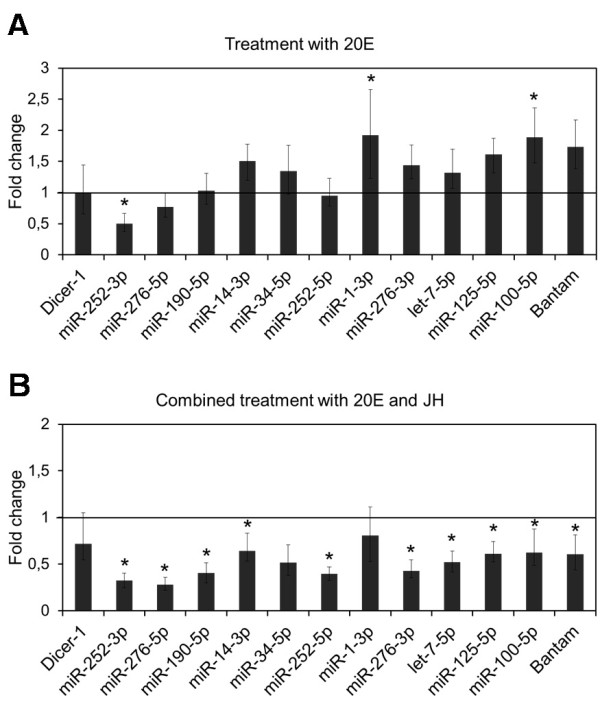
**Effect of 20-hydroxyecdysone (20E) and 20E plus juvenile hormone III (JH) on miRNA expression in *Blattella germanica*.** Specimens were treated with 1 μg of 20E (A) or with 1 μg of 20E plus 1 μg of JH (**B**) in freshly emerged last instar nymph and miRNAs were measured 24 h later. miRNA levels were measured by qRT-PCR, using U6 as a reference. Data represent 3 biological replicates (mean ± SEM) and are normalized against control females (reference value = 1); the asterisk indicates statistically significant differences with respect to controls (p < 0.05), according to the REST software tool [28].

To mimic the endocrine situation of a nymph-adult transition at the peak of 20E (like in N6), freshly emerged last instar female nymphs were treated with 1 μg of 20E. The results (Figure 3A) show that, statistically, most miRNAs were unaffected by the treatment. However, the expression levels of miR-1-3p and miR-100-5p were significantly increased after treatment with 20E, whereas those of bantam-3p, miR-125-5p, miR-14-3p, miR-276-3p, miR-34-5p and let-7-5p showed a tendency to increase with respect to controls, although differences were not statistically significant. The expression of *dicer-1* was not significantly affected by the treatment (Figure 3A).

On the other hand, the endocrine situation of a nymph-nymph transition at the peak of 20E (like in N5) was mimicked by treating equivalently staged specimens with 2 μg of JH and 1 μg of 20E. Results (Figure 3B) indicate that all the miRNAs studied, except miR-1-3p and miR-34-5p, had expression levels significantly lower than those of the controls, although the expression of the latter miRNA showed a tendency to be inhibited by the double hormonal treatment. The expression of *dicer-1* was not significantly affected by the treatment (Figure 3B).

### Functional analysis of miR-252-3p

**Figure 4:**
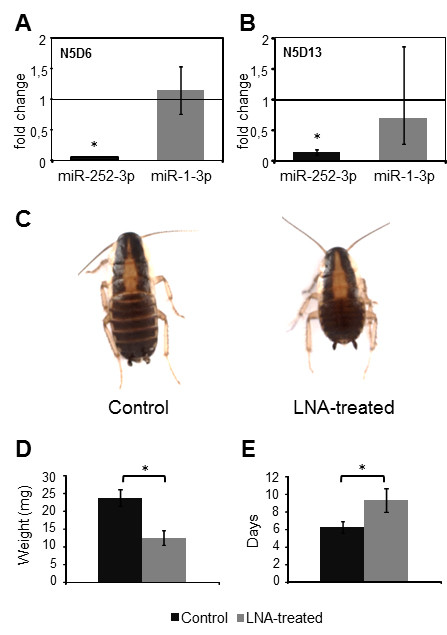
**Effects of miR-252-3p depletion on nymphal development of *Blattella germanica*.** Treated females received two injections of 50 μM of miR-252-3p LNA on day 1 and day 3 of fifth nymphal instar (N5D1 and N5D3, respectively); control females received an equivalent treatment with miRCURY LNA™ microRNA Inhibitor Negative Control A. **A**) Levels of miR-252-3p and miR-1-3p (used as negative control) on N5D6. **B**) Same data in N5D13. **C**) General aspect of a control specimen and a specimen treated with miR-252-3p LNA (LNA-treated) on N5D6. **D**) Weight of control and LNA-treated specimens in N5D6. **E**) Length (days) of N5 in control and LNA-treated specimens. qRT-PCR data in A and B represent 3 biological replicates and are normalized against the control females (reference value = 1); the asterisk indicates statistically significant differences with respect to controls (p < 0.05) according to the REST software tool [28]. Data in D and E represent 17 biological replicates; the asterisk indicates statistically significant differences with respect to controls (*t*-test, p < 0.01).

Depletion of miR-252-3p was achieved with two doses of 1 μL of miR-252-3p LNA at 50 μM each injected in N5, one 24 h after the moult (N5D1) and another 48 h later (N5D3). On day 6 of the instar (N5D6), the levels of miR-252-3p had specifically decreased (Figure 4A), and had slightly recovered seven days later (N5D13), although they were still significantly lower than the levels of the controls (Figure 4B). Specimens treated with miR-252-3p LNA grew slower than controls, as observed on day 6 (N5D6; Figure 4C). On N5D6, when controls moulted to N6, the weight of treated specimens was significantly lower than that of controls (Figure 4D) and they did not moult until 4–7 days later (Figure 4E). Although they were observed to grow more slowly, treated specimens still moulted to the next (N6) nymphal instar with no apparent defects, and then developed normally in N6 and moulted to the adult stage within the developmental time schedule of controls and with no apparent morphological defects. According to these results, miR-252-3p seems not regulate metamorphosis, but may be rather related with growth and development in penultimate nymphal stage.

## Discussion

High throughput sequencing of whole body miRNAs around the peak of 20E in the N5 and N6 libraries of *B. germanica* gave a total of 61 canonical miRNAs that were present in both libraries, although in different proportions in each. In a previous whole body miRNA library prepared during N6, but representing all eight days of the stage, we obtained a total of 49 canonical miRNAs [14]. Therefore, the number of miRNAs recovered in the present study, using animals collected around the peak of 20E, is higher. This suggests that most miRNAs might be more efficiently expressed around the peak of 20E, and that there was a dilution effect in the former library, which had all the days of the instar represented. Practically all canonical miRNAs reported by Cristino et al. [14] are present (except miR-1-5p and miR-184-5p), and are the most abundant in the present libraries. Some of the miRNAs (miR-8, miR-9a, miR-10, miR-71, miR-252, miR-276, miR-281) are represented by both strands, -3p and -5p, as was also observed in the previous N6 library [14].

The comparative analysis of N5 and N6 libraries allowed the identification of three and 37 miRNAs significantly more expressed in N5 and N6 respectively (Figure 1). Subsequently, 12 of these 40 miRNAs were investigated further by qRT-PCR to establish their expression patterns during N5 and N6. Results indicate that miR-252-3p is the only clear representative of miRNA that is well expressed in N5 and with decreased expression in N6, whereas let-7-5p, miR-100-5p and miR-125-5p are clear representatives of the reverse pattern (Figure 2). Whereas the expression pattern of miR-252-3p had yet to be studied in other insects, those patterns of let-7, miR-100 and miR-125 have been thoroughly examined. In *D. melanogaster*, these three miRNAs cluster in the same primary transcript, as first revealed by Bashirullah et al. [31] and Sempere et al. [30], and later confirmed in this fly and other insects [5,32]. Thus, the members of this miRNA cluster are expressed simultaneously, and studies in the holometabolan species, like *D. melanogaster* and *B. mori* have shown that expression concentrates in pre-metamorphic stages. In *D. melanogaster*, expression starts in late third (last) instar larvae, around the peak of 20E that triggers puparium formation, and continues until the imaginal moult [30,33]. In *B. mori*, let-7 expression starts in the moult leading to the penultimate larval instar, and continues until the emergence of the adult [34]. In the hemimetabolan *B. germanica*, the present study shows that expression of let-7, miR-100 and miR-125 concentrates in the last nymphal instar, having an expression burst around the peak of 20E that triggers metamorphosis (Figure 2).

Of note, among the miRNAs selected to study the expression pattern, we included miR-252 and miR-276, which were represented by both the -3p and -5p strands. In principle, the two respective strands originate from the same precursor and both should presumably be expressed at similar rates through parallel patterns. But this was not found to be the case. In the case of miR-252, the -3p strand is expressed at much lower levels than those of the -5p strand, whereas in the case of miR-276, the situation is the reverse; therefore, in each case the pattern of expression of one strand is clearly different to the expression of the other (Figure 2). These observations suggest that the expression is regulated differently in both strands, and that they play distinct miRNA functions [5]. In support of this notion, Liu et al. [35] reported that both strands of miR-276 are differentially expressed in different tissues and stages of *B. mori*, although miR-276-3p is always the most highly expressed strand.

Among the patterns examined, most of the expression peaks, either in N5 or N6, occur close to a peak of 20E (Figure 2), which suggests cause-effect relationships. This possible hormonal regulation of miRNA expression was therefore tested experimentally. In general, the experiments of hormonal treatments suggested that 20E alone tends to stimulate miRNA expression, whereas JH attenuates, or even inhibits the 20E stimulatory effect (Figure 3). Among those miRNAs that increased in expression after 20E treatment, we found let-7, miR-100 and miR-125. The increase in expression of miR-100-5p after 20E treatment is statistically significant, whereas let-7-5p and miR-125-5p had a tendency to increase (Figure 3A). This is consistent with previous studies in *D. melanogaster* that revealed that 20E increases the levels of these miRNAs [30,36] by inducing the expression of the polycistronic primary transcript that contains the let-7, miR-100 and miR-125 precursors [37]. In *D. melanogaster*, the burst of expression of let-7-5p in prepupae is important to ensure appropriate remodelling of the abdominal neuromusculature during metamorphosis [38]. Moreover, an independent work of Caygill and Johnston [39] showed that during *D. melanogaster* metamorphosis, the absence of let-7-5p and miR-125-5p results in temporal delays in the terminal cell-cycle exit in the wing, and in the maturation of neuromuscular junctions of imaginal abdominal muscles. The authors focused on the latter process by identifying the *abrupt* (*ab*) gene as a let-7-5p target, and by providing evidence that showed that let-7-5p regulates the maturation rate of abdominal neuromuscular junctions during metamorphosis by modulating *ab* expression [39].

Conversely, the significant stimulatory effect of 20E on miR-1-3p and the tendency of 20E to stimulate miR-34-5p that we observed in *B. germanica* contrasts with the results obtained in *D. melanogaster*, where 20E did not affect miR-1-3p and inhibited miR-34-5p [30]. We interpret these differences as reflections of different modes of regulation of these miRNAs in the two model species. The differences are also illustrated by the results obtained after JH treatment of *B. germanica*, which abolished the stimulatory effects of 20E on practically all miRNAs (Figure 3B), which was also the case for miR-34-5p. This contrasts again with the observations of Sempere et al. [30], which suggested that JH has a stimulatory effect on this miRNA in *D. melanogaster*.

Possibly the most interesting result of the experiments of hormonal treatments is that of miR-252-3p, which was the only miRNA that was inhibited by 20E alone (Figure 3A). Moreover, JH did not seem to counteract the 20E effect, as compared to other miRNAs (Figure 3B). Inhibition by 20E suggests that the decrease of miR-252-3p in N6, and the increase interrupted by a plateau around the 20E peak in N5 (Figure 2), might be due to the action of 20E. The other strand, classically considered the “mature” strand of miR-252, miR-252-5p, has been found in a number of insects [20], and its expression has been carefully studied in *B. mori*, where it shows a continuous high expression from the spinning larvae to pupal and adult stages [40]. Conversely, miR-252-3p has never been studied, nor has it been related with insect moulting and metamorphosis. It was for these reasons that we studied it functionally.

Depletion of miR-252-3p levels in the penultimate (N5) nymphal instar caused retarded growth and developmental delays within the instar (Figure 4). At the end, however, the treated insects were able to moult to the last (N6) nymphal instar and to the adult normally. Observed delays in N5 reached as long as 14 days in some cases, and levels of miR-252-3p measured in specimens on N5D13, which were presumably going to moult within the next one or two days, had somewhat recovered compared with levels measured on N5D6, although they were still significantly lower than levels of the miRNA of the controls (Figure 4). The data suggest that miR-252-3p regulates transcripts that are important for growth and development in N5, in the transition to the developmentally important N6 which precedes the imaginal moult. The fact that the treated specimens were finally able to moult to N6 and to the adult stage even with reduced levels of miR-252-3p, suggests that these levels, although still quite low, are above the operative threshold for this miRNA.

## Conclusions

1. The comparative analysis of a miRNA library of the penultimate nymphal instar (N5) with an equivalent library from the last instar nymph (N6) of *B. germanica* revealed 40 canonical miRNAs that are differentially expressed in each library.

2. In terms of counts, three of these miRNAs were found to be significantly more expressed in N5, whereas 37 were found to be more expressed in N6. qRT-PCR studies confirmed that miR-252-3p is well expressed in N5 but not in N6, whereas let-7-5p, miR-100-5p and miR-125-5p showed the reverse pattern.

3. Hormonal treatments indicated that 20E increases the expression of let-7, miR-100 and miR-125, but inhibits that of miR-252-3p.

4. The involvement of let-7, miR-100 and miR-125 in metamorphosis has been shown in other insects. Depletion of miR-252-3p in *B. germanica* caused growth and developmental delays, which suggests that this miRNA is involved in regulating these processes prior to metamorphosis.

## Competing interests

The authors wish to declare that they have no competing interests.

## Author’s contributions

MR studied the miRNA expression patterns, the effect of hormonal treatments and the function of miR-252-3p, and drafted the manuscript. AdH carried out the comparison of the miRNA libraries. XB conceived and coordinated the study and wrote the final manuscript. All authors read and approved the final manuscript.
